# Congenital midureteral stenosis in children: a 13-year retrospective study based on data from a large pediatric medical center

**DOI:** 10.1186/s12894-021-00916-2

**Published:** 2021-11-08

**Authors:** Zhaoyi Meng, Defu Lin, Guannan Wang, Yanchao Qu, Ning Sun

**Affiliations:** Department of Urology, Beijing Children’s Hospital Affiliated With Capital Medical University, National Center for Children’s Health, No. 56 Nanlishilu Rd, West District, Beijing, 100045 China

**Keywords:** Midureteral stenosis, Hydronephrosis, Congenital, Obstruction, Children

## Abstract

**Background:**

Midureteral stenosis is very rare in children and can cause congenital hydronephrosis. We report our experience treating children with congenital midureteral stenosis at our center, focusing on the differences in preoperative diagnosis and treatment compared with other congenital obstructive uropathies.

**Methods:**

We retrospectively reviewed the medical records of 26 children diagnosed with congenital midureteral stenosis at our center between January 2007 and December 2020, such as preoperative examination methods, intraoperative conditions, and postoperative follow-up results.

**Results:**

Of the 1625 children treated surgically for ureteral narrowing, only 26 (1.6%) were diagnosed with midureteral stenosis, including 15 infants and 11 children. Eighteen (69.2%) were boys, 13 (50%) were affected on the left side, and 23 (88.5%) had isolated ureteral stenosis. Overall, 13 (50%) of the children presented with prenatal hydronephrosis, and 13 (50%) presented with abdominal pain or a mass. All the children had undergone urinary ultrasound and intravenous urography preoperatively; the diagnostic rate of ultrasound was 92.3%. Only 7 (26.9%) children had undergone pyelography. All the children had undergone surgery. The ureteral stenotic segment was less than 1 cm long in 25 (96.2)% of the children. The mean follow-up duration was 22 months (range: 6–50 months). One child developed anastomotic strictures. Urinary tract obstruction was relieved in the other children without long-term complications.

**Conclusions:**

Congenital midureteral stenosis is rare, accounting for 1.6% of all ureteral obstructions, and its diagnosis is crucial. Urinary ultrasound has a high diagnostic rate and should be the first choice for midureteral stenosis. Retrograde pyelography can be used when the diagnosis is difficult, but routine retrograde pyelography is not recommended. Congenital ureteral stenosis has a relatively short lesion range, largely within 1 cm. The treatment is mainly resection of the stenotic segment and end-to-end ureteral anastomosis, with a good prognosis.

## Background

Congenital midureteral stenosis is characterized by a narrowing that occurs between the two ends of the ureter and is a very rare cause of hydronephrosis and dilatation of the upper ureter in children. In general, most upper urinary tract obstruction lesions are located at either the proximal or distal end of the ureter, such as ureteropelvic junction obstruction (UPJO), ureterovesical junction obstruction (UVJO) and nonrefluxing megaureter, and stenosis in the main length of the ureter is extremely rare. Campbell reported that, in an autopsy of a sequence of 12,080 children, 72 children had ureteral stenosis, 34% at the UVJ and 62% at the UPJ, and only 4% (3 children) had middle ureteral stenosis [1]. However, according to our statistics, middle ureteral stenosis accounts for only 1.6% of all ureteral strictures.

Because this condition is relatively rare, the diagnosis may be confused with UPJO or UVJO, particularly when ureteral dilation above the stenotic segment is not evident. Previous studies in the literature have reported low rates of preoperative midureteral stenosis diagnosis; this condition is usually found intraoperatively or by retrograde pyelography after anesthesia. Because our center is a large children’s medical center in northern China, many children with midureteral stenosis have been treated at our center over the past decade. We observed a relatively high rate of preoperative diagnosis of this condition, so we hypothesized that preoperative ultrasound and intravenous urography (IVU) are of great help to the diagnosis of midureteral stenosis. In this study, we describe our experience in treating children with midureteral stenosis, focusing on the preoperative diagnosis, surgical management and posttreatment outcomes.

## Methods

We reviewed the clinical records of children diagnosed with midureteral stenosis at Beijing Children’s Hospital from January 2007 to December 2020 (The basic characteristics of the children are shown in Table 1). Urinary ultrasound examination was performed by a professional sonographer with more than 5 years of working experience. Before all ultrasound examinations, the children fasted for 6–8 h and then drank sufficient water (more than 500 ml) over 30 min to induce diuresis and dilate the renal pelvis and ureter to enhance the detection of ureteral obstruction. At the time of examination, the child was first placed in the supine position and then on the side in the decubitus position. When the ureter showing expansion suddenly narrowed or was suddenly interrupted, careful observation was made to determine whether thickening of the ureteral wall or external oppression occurred in this position. Children with abdominal distension were treated with laxative suppositories to stimulate exhaust defecation to reduce the interference of intestinal flatulence. After the examination, the sonographer marked ureteral stenosis at the projection point on the body surface. Intravenous urography (IVU) was performed 10, 20 and 40 min after the contrast agent was injected.

**Table 1:** Patient clinical characteristics

Age (years)	
Infant	1.99 ± 0.72
Children	8.9 ± 2.9
Sex	
Male	18 (69.2%)
Female	8 (30.8%)
Side	
Left	13 (50%)
Right	13 (50%)
Stenosis	
Isolated	23 (88.5%)
Multiple	3 (11.5%)
Symptoms	
Prenatal hydronephrosis	13 (50%)
Abdominal/flank pain	11 (42.3%)
Abdominal mass	2 (7.7%)
Prior surgery	
Nephropyeloplasty	3 (11.5%)
Bilateral ureter reimplantation	1 (3.8%)
Nephrostomy	1 (3.8%)
Associated anomalies	
Contralateral multicystic dysplastic kidney	3 (11.5%)
Isolated kidney	2 (7.7%)
Dysrotation of renal axis	2 (7.7%)
Ipsilateral UPJO	2 (7.7%)

Magnetic resonance urography (MRU) was performed in 3 children, and computed tomography urography (CTU) was performed in 7 children. In children suspected of having multiple or long stenotic segments without obvious dilation of the proximal ureter and without a thick-thin junction of the ureter across the iliac vessels and in other cases with an unclear diagnosis, we performed preoperative retrograde pyelography after anesthesia. Iohexol and 0.9% saline at a ratio of 1:1 were selected for retrograde pyelography. If the child had a nephrostomy at another hospital, we used a direct renal fistula tube for antegrade pyelography. Voiding cystourethrography (VCUG) was performed to rule out vesicoureteral reflux (VUR) in 4 children with ultrasound or IVU indicating distal ureteral dilation or a history of fever that was not determined to be caused by urinary tract infection. Radioisotopic diethylenetriamine pentaacetic acid (DTPA) was used to calculate relative renal function and assess the degree of obstruction in 10 children (Table 2).

**Table 2:** Preoperative imaging studies

	Total	Positive	Negative
Urinary ultrasound (US)	26 (100%)	24 (92.3%)	2 (7.7%)
Intravenous urography (IVU)	26 (100%)	20 (76.9%)	6 (23.1%)
Computerized tomography urography (CTU)	7 (26.9%)	6 (85.7%)	1 (14.3%)
Magnetic resonance urography (MRU)	3 (11.5%)	3 (100%)	0 (0%)
Pyelography (antegrade or retrograde)	7 (26.9%)	7 (100%)	0 (0%)

The surgeries were performed by 5 pediatric urologists with 10 years of clinical experience. The open surgery used a transverse incision on the outer edge of the rectus abdominis muscle of the lower abdomen to expose the ureter and iliac vessels using an extraperitoneal approach. After removing the narrow segment, the distal end of the ureter was explored and a catheter was inserted into it for the water injection test to observe whether resistance was evident in the distal ureter. After confirming no obstruction at the distal end, ureteroureterostomy was performed. Laparoscopic surgery used a transperitoneal three-port laparoscope to remove the narrow ureter and perform an intracorporeal suture. All the resected segments were sent for pathological examination, and the diagnosis of ureteral stenosis was based on intraoperative observation and histological examination. Follow-up protocols included ultrasound every three months in the first postoperative year, every six months in the second, and every one to two years thereafter; intravenous pyelography or dynamic nuclide renal imaging was performed at 6 and 12 months postoperatively.

## Results

Over the past 13 years, 1625 children had received surgical treatment for ureteral obstruction at our center. Only 26 (1.6%) cases of hydronephrosis were caused by midureteral stenosis. Of these children, 18 (69.2%) were boys, 13 (50%) were affected on the left side, and 23 (88.5%) had isolated stenosis. Additionally, 15 were infants, with an average age of 1.99 ± 0.72 years and presenting primarily with prenatal hydronephrosis; 11 were children, with an average age of 8.9 ± 2.9 years and presenting primarily with intermittent abdominal pain or an abdominal mass. Among them, 5 children had received surgical treatment at another hospital and were referred to our hospital because of failure of the treatment to relieve the urinary obstruction. One of them was an 11-year-old girl who had bilateral hydroureter and was diagnosed at another hospital with right ectopic ureterocele and left side grade III VUR. She had undergone bilateral ureteral reimplantation. Six months after surgery, her right kidney hydroureter still existed, and she still had intermittent abdominal pain. The remaining 4 children had undergone pyeloplasty because of hydronephrosis, and the ureter was still obstructed after surgery. The renal fistula was maintained for 6 to 20 months when referred to our hospital and was not removed postoperatively. One of four children had undergone a second pyeloplasty and still had ureteral obstruction. No cases of urinary infection were found. According to the Society of Fetal Urology classification, 21 children had grade 3 hydronephrosis, 5 had SFU grade 4 hydronephrosis and obvious ipsilateral upper urinary tract obstruction, which was confirmed on DTPA imaging in 10 children and on IVU in the remaining 16, showing severe dilation of the renal pelvis and calyces. VCUG examination revealed contralateral grade I VUR in one child and grade III VUR in another.

**Fig. 1:**
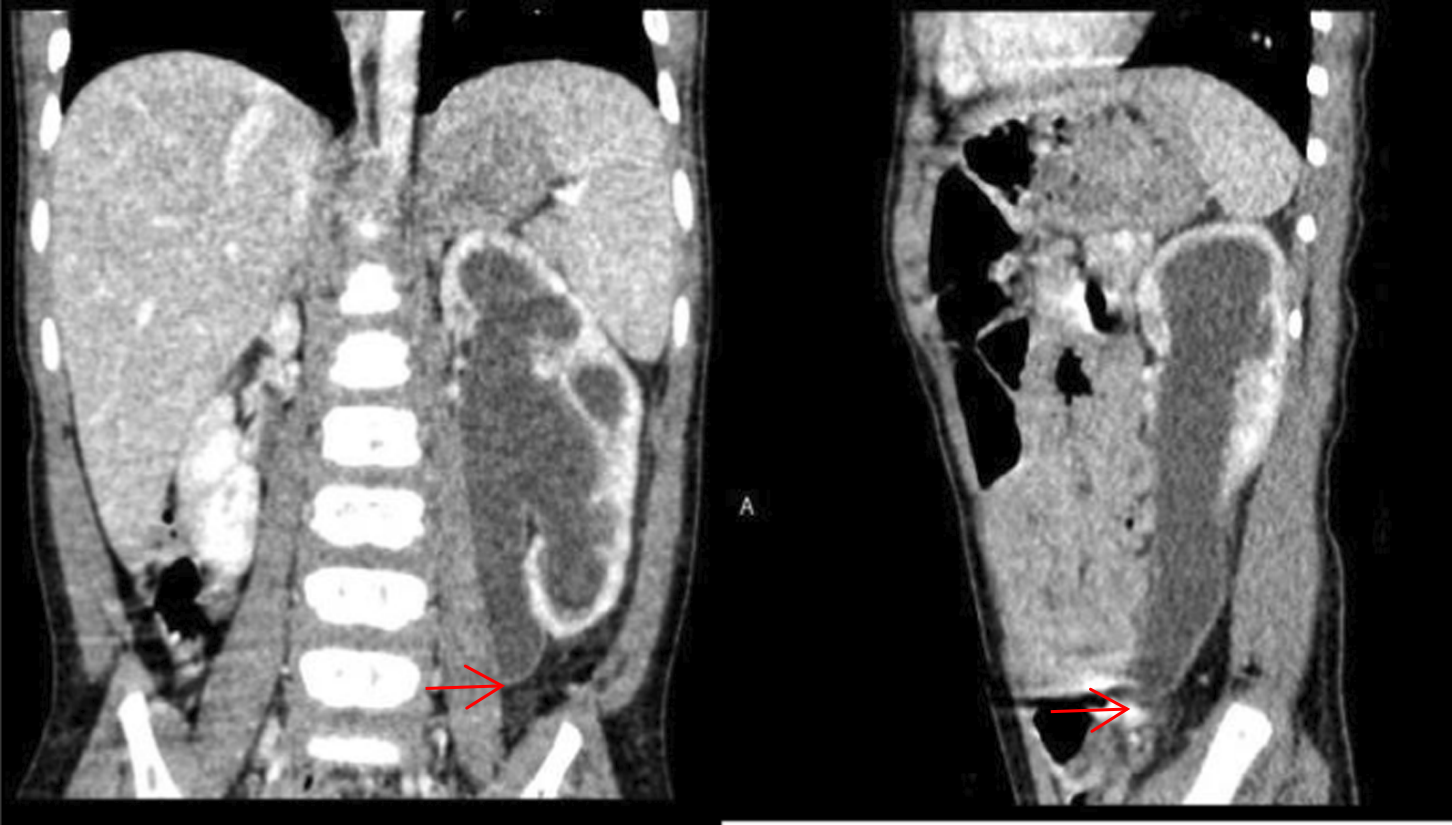
A 12-month-boy, CTU images on coronal and sagittal show dilation of the proximal ureter and midureteral stricture (arrow)

**Fig. 2:**
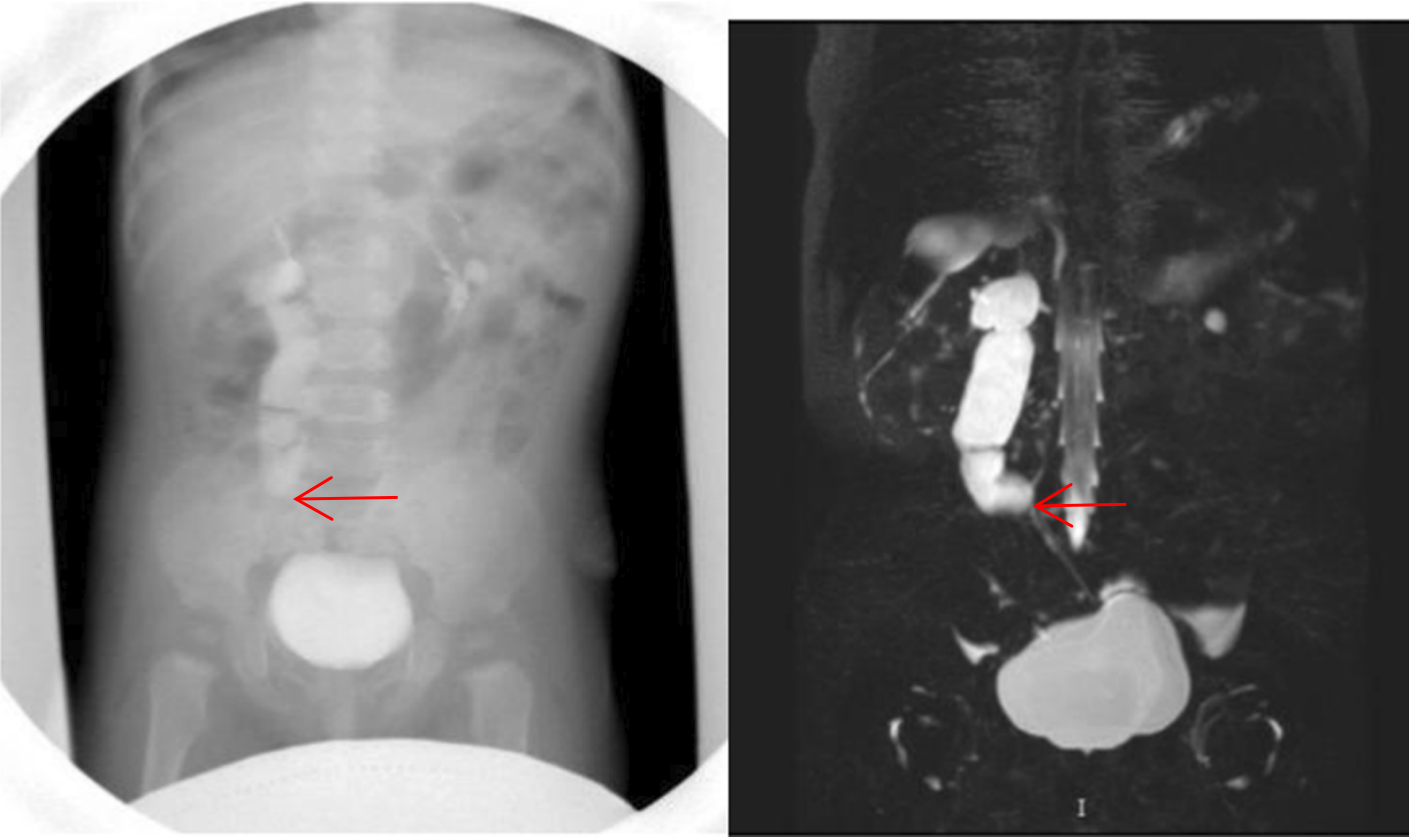
A 8-year-old boy with midureteral stenosis (arrow), IVP and MRU images show the left proximal ureter turning medial and tapering, with proximal ureter dilation

**Fig. 3:**
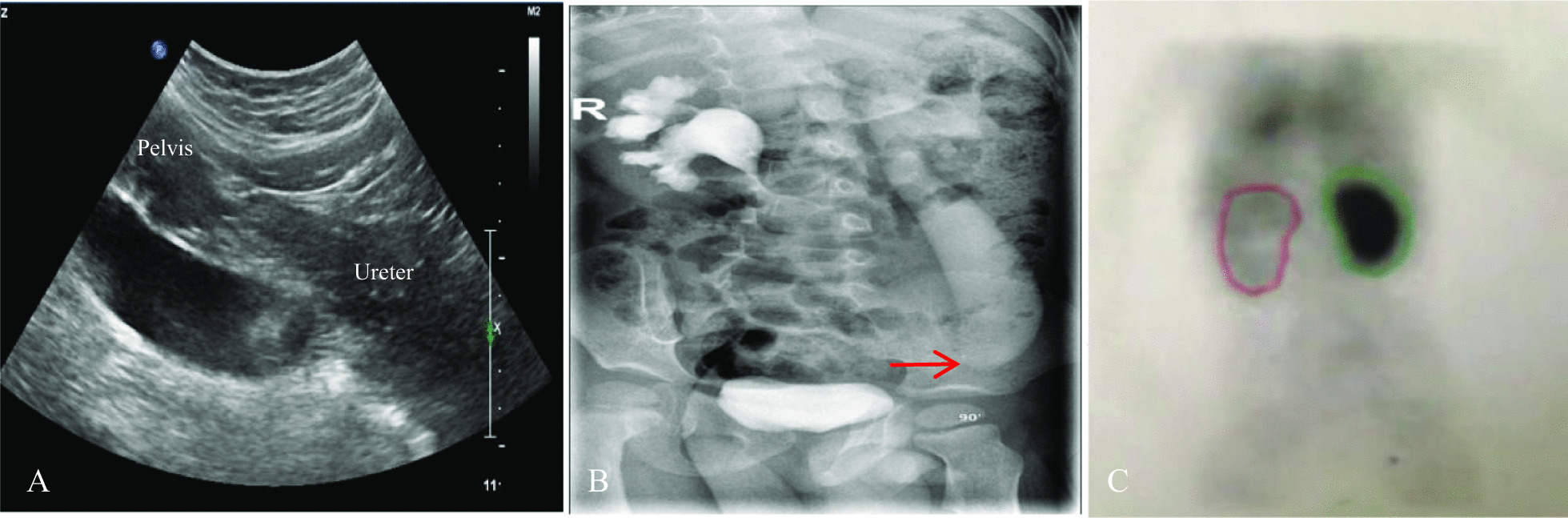
A 1.2-year-old boy. **a** Ultrasound of the affected kidney shows hydronephrosis and hydroureter. **b** IVU reveals the left midureteral stenosis (arrow); **c** preoperative DTPA renography demonstrated delayed drainage of the left kidney

Preoperative assessment was performed in all children using IVU and urinary ultrasound. Preoperative ultrasound revealed 24 cases of midureteral stenosis and 2 cases of UVJO, for a diagnostic rate of 92.3%. Midureteral stenosis was confirmed in 9 cases by ultrasound and IVU, in 9 cases by MRU or CTU, in 7 cases by antegrade or retrograde pyelography (1 case through a renal fistula, 2 cases by percutaneous pyelography and 4 cases by retrograde pyelography), and in 1 case by intraoperative diagnosis (Figs. 1, 2 and 3; Table 2).

**Fig. 4:**
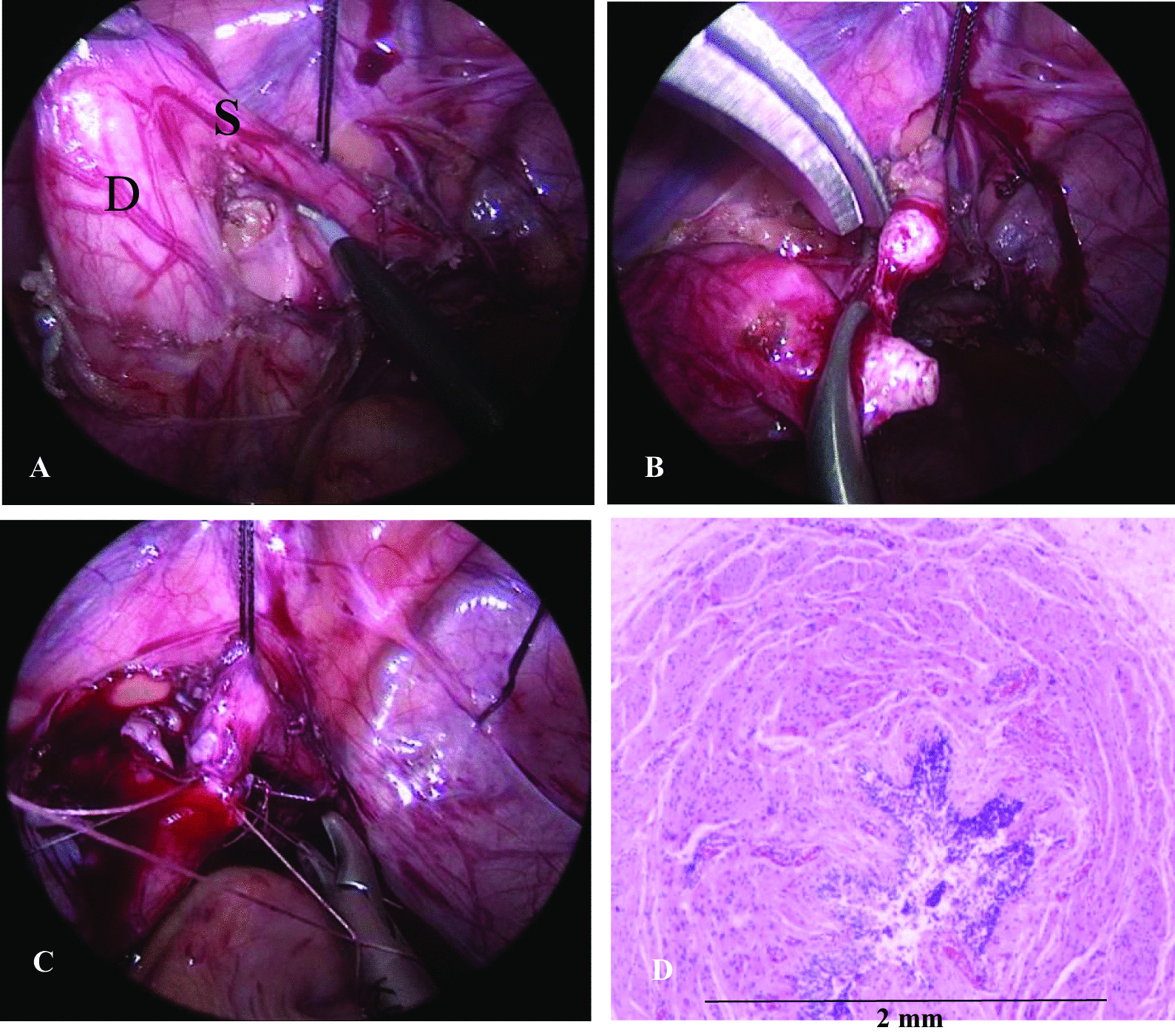
A 3-year-old boy. **a** Congenital midureteral stenosis (S) and dialated proximal ureter (D). **b** Ureterectomy of the stenosed segment. **c** Ureteroureterostomy. **d** Pathology of the resected segment showing ureteral lumen stenosis

While open surgery was used in most of our cases, laparoscopic surgery was performed in 3 cases (Fig. 4). In 23 (88.5%) cases, the stenotic site was located at or near the level of the iliac vessels. The stenotic segment was less than 1 cm long in 25 (96.2%) of the children, and no stenotic segments were longer than 2 cm. Therefore, for a single site of ureteral stenosis, 22 (84.6%) patients had undergone ureteroureterostomy. Tension-free anastomosis was achieved in these children. A 10-year-old boy was diagnosed with middle ureteral stenosis and ipsilateral multicystic dysplastic kidney (MCDK). Preoperative renal dynamic imaging showed that the differential renal function was approximately 9%, and the proximal ureter was expanded by approximately 2.5 cm, but the middle ureter was not significantly expanded. Because of suspected multiple ureteral stenoses, retrograde pyelography was performed after anesthesia, and multiple ureteral stenoses were confirmed in the upper and middle ureters. The child had undergone nephroureterectomy after approval was obtained from his parents. The 11-year-old girl’s ureteral stenosis was approximately 1.5 cm in length. After this stenosed segment was removed, anastomosis of the two broken ends of the ureter could not be completed. The proximal end of the ureter was measured to be approximately 5 cm from the bladder, so a Boari flap was used for anastomosis. Ileal ureteral replacement was used in two children with multiple ureteral strictures. The stenotic ureter was replaced with 12 cm and 15 cm isoperistaltic ileum intestinal segments. The characteristics of the stenotic segment and surgical methods are shown in Table 3.

**Table 3:** Characteristics of and surgical technique used for the stenosis segment

Stenosis location	
At or near the iliac vessels	23 (88.5%)
Scattered multiple stenosis	3 (11.5%)
Length of the stenosed segment	
L ≤ 5 mm	17 (65.4%)
5 mm < L ≤ 10 mm	8 (30.8%)
> 10 mm	1 (3.8%)
Surgical technique	
Ureteroureterostomy	22 (84.6%)
Boari bladder flap	1 (3.8%)
Ileal ureteral replacement	2 (7.7%)
Nephroureterectomy	1 (3.8%)

Pathologically, the ureter was shaped similar to an umbrella or dumbbell on gross observation, with a narrow lumen in the middle through which the probe could pass but not urine. Microscopic examination of the stenotic area suggested submucosal fibrous tissue hyperplasia with scattered lymphocytic infiltration and muscularis thickening with muscle fiber degeneration (Fig. 4). All cases of ureteral stenosis were considered congenital, without an intrinsic or extrinsic pathology, and all the specimens were positive on Masson staining. Ureteral valves or folds were not identified in the longitudinal sections of specimens from any of the children. Associated anomalies were observed in 8 children, such as MCDK in 3 cases, an isolated kidney in 2 cases, ipsilateral UPJO in 2 cases, and concomitant ipsilateral dysrotation of the renal axis in two cases. The details of the clinical characteristics of all the patients are shown in Table 1.

The total follow-up period ranged from 6 to 50 months (mean: 22 months). All 26 children were followed up by telephone, and 20 of them regularly visited our hospital for ultrasound examination. All the children were relieved of their symptoms, and none had palpable masses, abdominal pain or urinary infections. Postoperative ultrasound evaluation during the follow-up period showed a significant decrease in hydronephrosis in all the children. The average anteroposterior diameter of the renal pelvis (APD) of all the children was reduced postoperatively. Among them, 21 children with hydronephrosis SFU3 showed a decrease in the APD from 3.12 ± 0.05 mm before the operation to 2.35 ± 0.09 mm, and four children with SFU4 showed a decrease in the APD from 4.15 ± 0.19 mm preoperatively to 2.96 ± 0.16 mm. IVU showed no obstruction in the urinary tract of all the children. Eight children had undergone dynamic renal nuclide imaging, indicating that split renal function improved or stabilized after surgery. No electrolyte disturbances or VURs were found in the two children who had undergone ileal ureteral replacement. The contralateral kidney function of the boy who underwent nephroureterectomy was normal. The child who had undergone Boari flap replacement of the ureter developed anastomotic stricture one year after surgery that was resolved after a third surgical procedure. No other complication occurred.

## Discussion

Congenital middle ureteral stenosis is a rare cause of hydroureteronephrosis. To date, few studies have investigated the incidence of ureteral stenosis, and the previously reported incidence is based on a large-scale autopsy of children. We reviewed the clinical data of 1625 children who had undergone surgical treatment because of an obstructive hydroureter in the past 13 years. Among them, 26 had congenital midureteral stenosis, accounting for approximately 1.6%, which was lower than that reported in the literature. However, our statistical data were based on actual clinical data and were more representative. The pathogenesis of this condition is unclear, and many theories attribute it to abnormal embryonic development, including abnormal fetal vessel compression, intrauterine inflammation, incomplete ureteral recanalization, ischemia due to abnormal branches of blood vessels and localized developmental arrest [1–5]. A review of the previous literature has revealed that in children, congenital midureteral stenosis is often associated with urological anomalies, such as contralateral renal agenesis or atrophy, VUR, UPJO, crossed renal ectopia, solitary kidney and contralateral blind-ending ureter [6–8], suggesting the possibility of bilateral aberrant renal and ureteral development and indicating that ureteral stenosis may be a mild manifestation of unilateral hypoplasia. In the present study, one case of contralateral MCDK with systemic growth retardation and two cases of contralateral renal axis malrotation were found.

Pathologically, ureteral stenosis is a mechanical obstruction due to structural abnormalities in the wall that are distinct from the neurogenic and myogenic mechanisms of UPJO. Two etiologies of ureteral stenosis exist: ureteral valves and true ureteral stenosis. Ureteral valves are anatomically demonstrable transverse folds of ureteral mucosa, potentially containing bundles of smooth muscle fiber covered with normal urothelium [9]. In the present study, no ureteral valves were found in any of the children after careful observation of the pathological specimens. Studies on the ultrastructure of ureteral stenosis found that the stenotic ureter did not deviate fundamentally from its pattern, and only quantitative changes in its composition were observed. These changes included lumen shrinkage and relative or absolute loss of smooth muscle with a normal, altered or disorganized arrangement, with or without connective tissue changes [1–8]. Our pathological specimens exhibited smooth muscle hyperplasia and fibrous tissue degeneration. A low level of chronic inflammatory cell infiltration was observed in the narrow segment, a finding that is consistent with that reported in the literature.

Midureteral stenosis occurs at the level of the bifurcation of the common iliac vessels, also a site of physiological ureteral narrowing. In this study, stenosis in this region accounted for 88% of cases. Ninety-six percent of the stenotic segments were within 1 cm in length, and upper ureteral stenosis was more common in patients with multiple stenotic sites and was rarely isolated. Additionally, ureters with multiple ureteral stenotic sites were generally poorly developed and small in appearance. Previous literature has demonstrated that at sites of stenosis, the ureter lumen usually shrinks by approximately 60% and significantly impedes urine delivery [6]. However, clinically, we found that compared with UPJO, children with midureteral stenosis had relatively mild hydronephrosis and a later onset of symptoms. In this study, 12 children were older than 5 years when they developed symptoms. Midureteral stenosis has also been diagnosed at 15 and 20 years of age [10, 11]. The upper ureteral dilation in some children was not very severe, likely because of absorption by ureteral lymphoid tissue and the buffering effect of the ureter on the urine during low stenosis. Murnaghan speculated that the narrow segment transmitted peristaltic waves when the urine flow was low, allowing smooth passage; when the urine flow load exceeded a critical value, the narrow segment decompensated, resulting in clinical symptoms [12].

Midureteral stenosis results in dilatation of the ureter above the stenotic site, usually with dilatation of the renal pelvis. The ureter may also stretch, curl and droop clinically similar to that of UVJ malformation. However, these two diseases are very different. Midureteral stenosis comprises an obstruction at a certain distance from the bladder; in intramural ureteral lesions, the ureter is usually dilated next to the bladder. Previous studies have shown that the preoperative diagnosis of midureteral stenosis is relatively difficult because of its symptomatic and radiographic similarity to intrinsic UVJO or UPJO. Most cases are diagnosed intraoperatively or by retrograde pyelography, and the rate of diagnosis on preoperative ultrasound is low. Many scholars have considered ultrasound to have limited value in distal ureteral obstruction localization. Hawang et al. believed that children with hydronephrosis and megaureter should routinely undergo retrograde pyelography unless the distal ureter is well demonstrated on other tests [1]. MRU is recommended to be routinely performed in children with ureteral dilatation because MRU provides excellent anatomic and functional details of the collection system, allowing the accurate diagnosis and treatment of ureteral stenosis [13]. However, routine retrograde pyelography in children with congenital hydronephrosis is controversial. It is an invasive examination with the risks of ureteral injury, urinary tract infection, and iatrogenic obstruction of the UVJ. Ruston pointed out that retrograde pyelography or contrast visualization of the ureter before pyeloplasty is rarely necessary in children [14]. Previously, Cockrell et al. reported that the secondary abnormalities found by retrograde pyelography were in the surgical field where they would be detected and managed normally [15]. The main purpose of RGP or MRU examination is to clearly show the shape of the ureter and clarify the location and number of ureteral stenoses. However, performing RGP or MRU is not required routinely but is critical for secondary surgery and in children with unclear diagnoses.

With the development of ultrasound techniques for ureteral examination and improvements in the diagnostic rate, ultrasound can serve as a noninvasive and repeatable effective method to diagnose ureteral stenosis or stricture. Ultrasound can be used to determine the location of a ureteral obstruction according to the degree of ureteral dilation and morphological changes, as well as to determine the etiology and extent of stenosis according to differences in the acoustic images of the stenosis site. At our center, the diagnosis of ureteral stenosis mainly relies on ultrasound, IVU and CTU, while MRU and retrograde pyelography are performed irregularly. The accuracy of the ultrasound examination is highly dependent on the personal experience of the examiner. Notably, our rate of preoperative midureteral stenosis diagnosis is higher than that reported in the literature, with the diagnostic rate of ultrasound reaching 96%. As early as 10 years ago, related reports in the domestic literature demonstrated rates of coincident lesion location diagnosis and quantitative ureteral stricture diagnosis by ultrasound of 96.6% and 91.5%, respectively [16]. The ultrasound examination and diagnosis at our center were completed by sonographers with certain clinical experience in urology. It is a real-time dynamic examination accompanied by parents. Before the examination, the children had fasted for approximately 6 h and drank adequate water to maintain a state of bladder filling. In the state of holding urine, the prone sites of ureteral stenosis were examined emphatically and repeatedly—namely, the ureteropelvic junction, vicinity of ureter crossing iliac vessels and vesicoureteral junction. Ureteropelvic junction obstruction combined with ureterovesical junction obstruction and iliac ureteral vessel stenosis is very rare; but for children with hydronephrosis, exploring the ureter repeatedly by ultrasound is necessary, particularly the three common sites of ureteral stenosis and those with hydronephrosis. Next, after allowing the child to urinate, another examination was performed and compared with the first examination. Because of the sound window of the kidney and bladder, the UPJ and terminal ureter adjacent to the bladder were well displayed; however, the ureter in the middle segment was deep, and the ureter did not dilate behind the bladder in many cases. Therefore, ureteral stenosis can be missed or a dilated bowel can be mistaken for a ureter. To avoid these situations, we prepared the bowel sufficiently to reduce interference with intestinal contents and gases. When necessary, the children were placed in the prone position and padded with cotton pads with a height of approximately 15 cm on the upper abdomen to naturally increase abdominal pressure and then checked again. We believe that the false-negative rate is due to limited experience; an experienced sonographer can diagnose most cases of midureteral stenosis.

IVU is a common and accurate visual examination to diagnose ureteral stenosis. If children have good renal function, IVU can often clearly show the degree of ureteral hydronephrosis and shape and location of ureteral stenosis, and the accuracy of ureteral stenosis localization is high. Long-term obstruction causes poor renal function, which must be extended to approximately 1 h to improve the positive rate of diagnosis. Therefore, we do not recommend preoperative MRU or retrograde pyelography in children with suspected midureteral stenosis but instead propose ultrasound and IVU for routine preoperative evaluation. When ureteral dilatation is not obvious, the distal ureter is not clearly displayed, or the diagnosis is unclear, such as suspected cases of multiple stenotic sites, MRU, CTU or retrograde pyelography will be performed to confirm the diagnosis.

Preoperative ultrasound localization is an essential reference surgical approach selection. The surgical method is considerably affected by the location and number of stenotic segments. Most single stenotic segments are short. Thus, resection of the stenotic segment and ureteroureterostomy are feasible. For upper ureteral stenosis combined with UPJO, pyeloplasty can be performed directly. In two children with multiple left ureteral stenoses, because the stenosis was relatively dispersed and the distance was longer, we adopted ileal ureteral replacement. Technically, replacing the left ureter with the appendix is challenging because the appendix in children is short and the mesoappendix length is limited. Thus, the cecum and even the ascending colon must be moved to provide a sufficient vascular pedicle length, which is prone to postoperative complications such as poor blood transport, appendix necrosis, anastomotic stenosis, and intestinal obstruction. We did not use the appendix for left ureteral replacement. Laparoscopic and robotic technology has been successfully used to treat ureteral stenosis. Compared with open surgery, minimally invasive surgery has the advantages of less postoperative pain, a shorter hospitality stay, less scarring, and less parental anxiety. In the future, more children will undergo treatment with laparoscopic and robotic surgery. The prognosis of the patients in the present study was very good; the symptoms resolved on long-term follow-up, ultrasound examination suggested that hydronephrosis was relieved or resolved, the urinary tract was unobstructed, and IVU examination showed no obstruction.

The present study has some limitations. Because of the single-center retrospective design over a long period, the preoperative and postoperative clinical data of some children cannot be followed up, especially the treatment information of the 5 referred children in other hospital. Additionally, the renal dynamic imaging technology and level were limited, the postoperative follow-up of the children was primarily performed using IVU and ultrasound, and an accurate assessment of renal function was lacking. Additionally, the ultrasound examination technology introduced must be further summarized and promoted and does not apply to all hospitals.

## Conclusion

Congenital midureteral stenosis is a rare cause of hydronephrosis. A precise preoperative diagnosis is mandatory for successful surgical management. Ultrasound should be the first choice to examine ureteral stenosis in children. The combination of ultrasound and IVU has complementary advantages to improve the diagnostic accuracy. When a diagnosis is challenging or uncertainty in the ultrasound or IVU, further MRU or retrograde paragraph examination is necessary.

## Data Availability

The datasets used in the current study are available from the corresponding author on reasonable request.
